# Interventional oncology and immunotherapy: current status and future perspectives

**DOI:** 10.3389/fimmu.2025.1541105

**Published:** 2025-04-08

**Authors:** Ji Ma, Zhigang Wei, Xin Ye

**Affiliations:** ^1^ Department of Oncology, Lung Cancer Center, The First Affiliated Hospital of Shandong First Medical University and Shandong Provincial Qianfoshan Hospital, Shandong Lung Cancer Institute, Jinan, China; ^2^ Cheeloo College of Medicine, Shandong University, Jinan, China

**Keywords:** interventional oncology, immunotherapy, ablation, transarterial embolization, outcome

## Abstract

Interventional oncology has become an important part of multidisciplinary cancer treatment following the development of interventional radiology. Tumors can release antigens, activate immunity, and cause an abscopal effect after interventional therapy. However, the activated immune response is limited and involves a complex process. New methods to solve the problems were developed following the advent of immunotherapy. The combination therapies enhanced the antitumor immune response and improved patient outcomes with good application prospects. In this review, we have summarized the interventional therapies used to improve immune efficacy and discussed the advancements in combining interventional therapy and immunotherapy.

## 1 Introduction

Interventional radiology (IR) is a medical specialty that provides diagnostic and therapeutic procedures that are minimally or super minimally invasive. IR started in 1950s, with the introduction of the Seldinger technique, which allowed for the coaxial replacement of smaller-bore needles and catheters (1). In recent decades, IR has expanded considerably, transitioning from a technical subspecialty of radiology to encompass almost all aspects of patient care, and has gained independent professional status in many countries (2).

IR in oncology is termed as interventional oncology (IO). It combines the techniques of IR, enabling the diagnosis, treatment, and management of issues associated with cancer (3). With the exponential growth of local and regional percutaneous ablation treatment options in the late 1990s, notably in liver malignancies (4, 5), IO revolutionized the modern cancer treatment pattern and became a critical component of multidisciplinary oncology teams (6). The development of image guidance technologies, encompassing catheter angiography–based interventions, CT-guided puncture positioning techniques, and gadolinium-based magnetic resonance aortography, have also accelerated the expansion of IO (7), with increasing evidence suggesting that IR-directed procedures, such as tumor ablation, are comparable to surgical resection (8).

**Figure 1:**
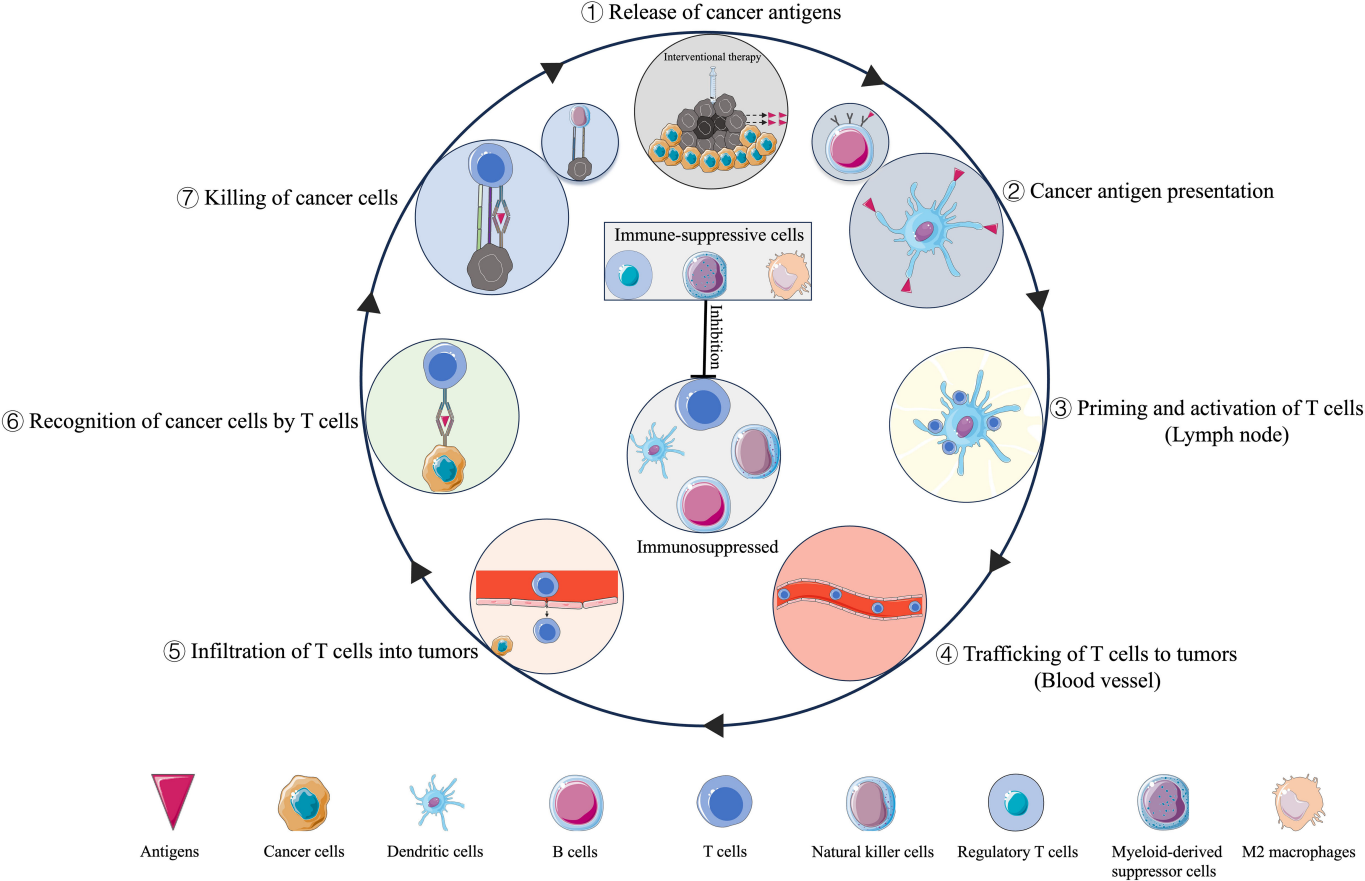
The seven steps of immunity circle in interventional oncology.

Although the interventional techniques and mechanisms of induced cell death are diverse, they share one key feature: *in situ* tumor destruction can provide an antigen source for the immune system (9). The released cancer antigens enter seven steps of the cancer-immunity cycle and kill the cancer cells (10) (**Figure 1**). The intervening material, released by tumor cells following intervention, mainly includes tumor antigens, heat shock proteins, and cytokines, can also induce antitumor responses distant to the targeted area, known as the abscopal effect (11). However, the activated immune response is limited and complex.

Immuno-oncology is an innovative cancer research and practice area that seeks to help the patient’s immune system fight cancer. The advent of immune checkpoint inhibitors (ICI), such as anti-programmed death-1 (PD-1) and its ligand PD-L1 or cytotoxic T-lymphocyte associated protein-4 (CTLA-4) inhibitor, represents an exciting time for interventional oncologists. During tumor cell death induced by interventional techniques, damage associated molecular patterns (DAMPs) and antigens that activate the immune system can be released, the process called the immunogenic cell death (ICD), it may sensitize patients to the subsequent ICI (12), and combination therapies may substantially improve patient outcomes. Other treatment methods that enhance immune responses, such as adoptive cell therapy (13), cancer nanovaccines (14), and novel biomaterials (15), have also shown good efficacy and promising outcomes.

**Figure 2:**
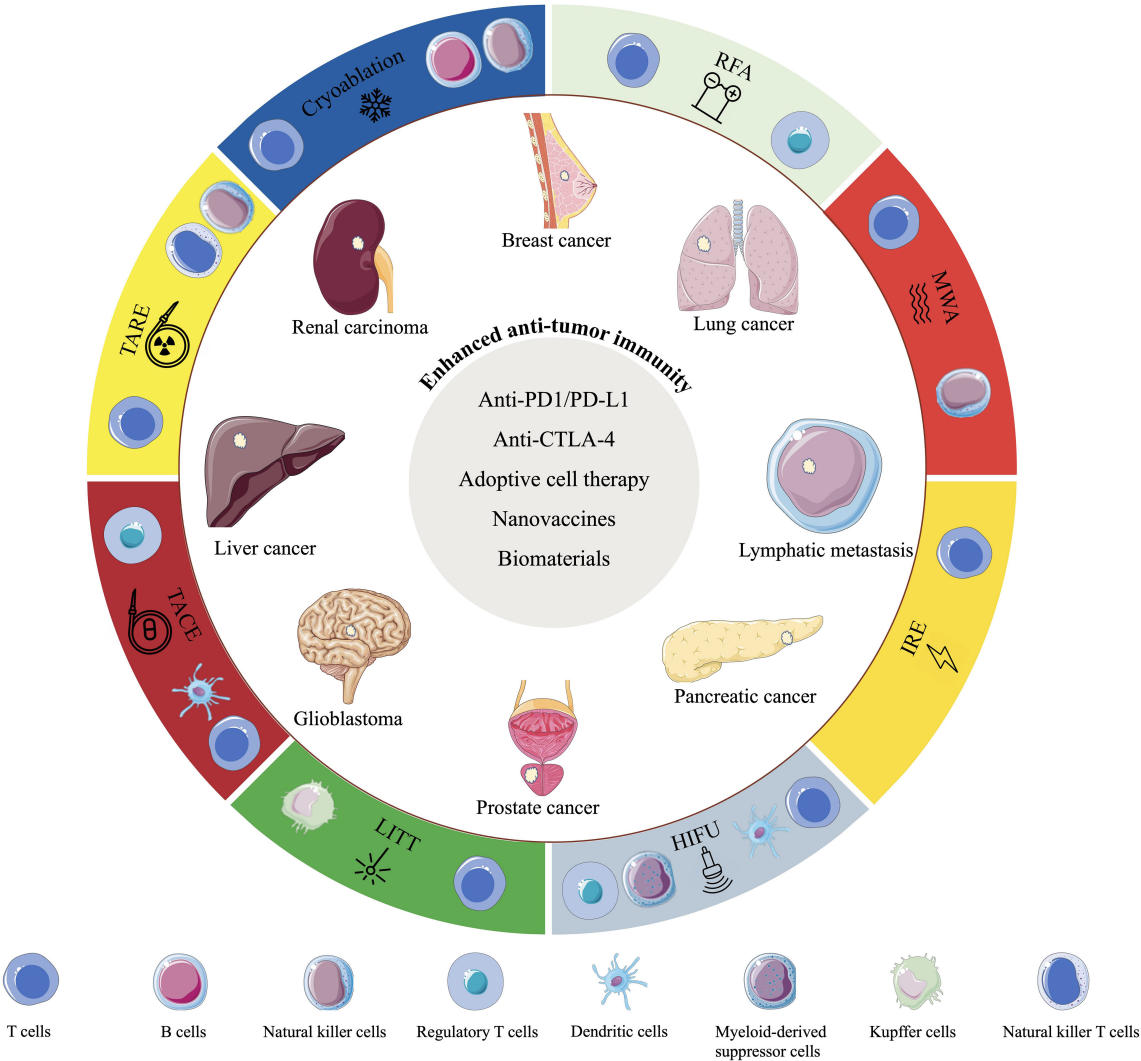
Schematic diagram showing the interventional oncology techniques, the changes in immunocyte types induced by these techniques, their application to common cancer types, and the combination therapies that enhance anti-tumor immune efficacy.

This review aims to summarize the applications of IO in the era of immunotherapy (**Figure 2**).

## 2 Ablation therapies and immuno-oncology

In contemporary precision cancer treatment, micrometastasis may be responsible for tumor recurrence after local treatment. Ablation therapies such as cryoablation, radiofrequency ablation (RFA), and microwave ablation (MWA) have been shown to induce systemic antitumor immune responses (16). Similar to the abscopal effect (11), these activated immune cells may contribute to the elimination of micrometastases. However, ablative therapy alone causes weak or even counterproductive immune effects (17), but when combined with immunotherapy, it could achieve amplified and sustained antitumor immune responses (18, 19). Ongoing trials on ablation therapies combined with immunotherapy are listed in **Table 1**.

**Table 1:** Ongoing trials of ablation therapies combined with immunotherapy.

NCT number	Enrollment	Phase/N	Design	Primary Measures	Study Completion
NCT06246968	Metastatic/Locally advanced TNBC	Phase 1 N = 30	Pembro + Cryo Pembro	6-week immune response	January, 2027
NCT03546686	TNBC	Phase 2 N = 80	Pembro + Cryo + Surgy + Pembro Ipi + Nivo + Cryo + Surgy + Nivo	3-year EFS	June, 2028
NCT05781074 NCT05893056 NCT05897268 NCT06032845 NCT05835245 (CASTLE-08,09,10,11, ICC-Chemo-free)	Advanced BTC Previously treated gastric cancer liver metastasis Advanced HCC Previously treated solid tumors Advanced ICC	Phase 2 N = 25 N = 30 (ICC-Chemo-free)	Cryo + Tisle + Len Cryo + Sint + Len (08)	2-year ORR	December, 2025
NCT04701918	Urothelial carcinoma	Phase 2 N = 30	Pembro/Avelu + Cryo	2-year ORR	December, 2025
NCT04299581	Advanced HCC	Phase 2 N = 25	Cryo + Camre	2-year ORR	May, 2024
NCT05779423	Metastatic melanoma resistant to PD-1 inhibition	Phase 2 N = 37	Cryo + Ipi + Nivo	6-month clinical benefit rate	January, 2025
NCT04339218	Metastatic lung adenocarcinoma	Randomized Phase 3 N = 214	Cryo + Pembro + CHT Pembro + CHT	1-year OS rate	August, 2025
NCT04864379	Advanced solid tumors	Phase 1 N = 30	RFA + PD-1 inhibitor + iNeo-Vac-P01	1-year AEs 2-year IFN-γ 3-year CD4/CD8 5-year ORR	August, 2025
NCT04727307	Small HCC	Randomized Phase 2 N = 202	Atezo + RFA + Atezo + Beva RFA	2-year RFS	February, 2031
NCT04990609	PDAC	Phase 2 N = 60	EUS-RFA + NAC	Complete number	May, 2028
NCT06190782	Oligometastatic ESCC	Randomized Phase 3 N = 354	PD-1 inhibitor + /- CHT + Local therapy (Radiotherapy, Surgery, RFA/MWA) PD-1 inhibitor + /- CHT	3-year PFS	September, 2027
NCT04952272	Advanced solid tumors	Phase 1 N = 50	MWA/DEB + CpG-ODN + /-CAR-T	3-year safety, efficacy	June, 2036
NCT05053802	Multiple primary lung cancer	Phase 2 N = 146	MWA + Camre MWA	1- and 3-year RFS 5-year lesions PFR	September, 2027
NCT03769129	NSCLC (IIIB-IV) failed with first-line therapy	Randomized Phase 3 N = 100	MWA + Pembro	2-year OS	November, 2029
NCT06248554	Early-stage HCC	Prospective cohort N = 200	MWA + PD-1 inhibitor	60-month DFS	December, 2024
NCT06205849	Locally advanced pancreas cancer	Phase 1 N = 18	IRE + Mitaza	12-week AEs	August, 2029
NCT06378047	Locally advanced pancreatic cancer	Phase 2 N = 3	IRE + Pembro	90-day AEs	April, 2027
NCT03080974	Advanced pancreatic adenocarcinoma	Phase 2 N = 10	IRE + Nivo	100-day AEs	April, 2026

TNBC, triple-negative breast cancer; Pembro, pembrolizumab; Cryo, cryoablation; Ipi, ipilimumab; Nivo, nivolumab; EFS, event-free survival; ICC, intrahepatic cholangiocarcinoma; BTC, biliary tract cancer; HCC, hepatocellular carcinoma; Tisle, tislelizumab; Sint, sintilimab; Len, lenvatinib; ORR, objective response rate; Avelu, avelumab; Camre, camrelizumab; PD-1, programmed death-1; CHT, chemotherapy; OS, overall survival; RFA, radiofrequency ablation; iNeo-Vac-P01, neoantigen cancer vaccine; AEs, adverse events; IFN, interferon; HCC, hepatocellular carcinoma; Atezo, atezolizumab; Beva, bevacizumab; RFS, recurrence-free survival; PDAC, pancreatic ductal adenocarcinoma; EUS, endoscopic ultrasound; NAC, neoadjuvant chemotherapy; ESCC, esophageal squamous cell carcinoma; MWA, microwave ablation; PFR, progression-free rate; NSCLC, non−small cell lung cancer; DFS, disease-free survival; DEB, drug-eluting beads; IRE, irreversible electroporation; Mitaza, mitazalimab (CD40 antibody).

### 2.1 Cryoablation

Modern cryotherapy began in the 1960s when a neurosurgeon developed a modern cryosurgical system using a probe to treat brain tumors and movement disorders (20). Cryoablation is a hypothermic modality that induces tissue damage via a freeze-thaw process.

The cryoablation technique utilizes the Joule-Thomson effect by releasing compressed liquid gases into target tumor tissues via specialized cryoablation probes. As the compressed liquid gas rapidly expands and transforms into the gaseous state, it produces an ultra-low temperature, rapidly decreasing the target tissue temperature to about −140°C. Mechanisms of cell death initiated by cryoablation include direct cell damage caused by ice crystal formation, microcirculatory failure after thawing, and induction of apoptosis and necrosis (21).

Compared to other ablation techniques, cryoablation offers superior precision in controlling the freezing range, enabling selective tissue damage while minimizing collateral effects on surrounding nerves and blood vessels. The low-temperature environment not only reduces the risk of thermal injury, but also promotes faster post-ablation healing. Cryoablation preserves the native antigenic structure and exhibit the highest potential to elicit post-ablation immunogenic responses, it generates a more robust immune response than other ablation therapies (22).

However, cryoablation involves a longer duration because sufficient time is required for the cryogen to fully act on the target area, a process that may take several hours; the low temperatures may lead to platelet depletion, increasing the bleeding risk; thus, patients with poor coagulation function should avoid this treatment method. The debate persists over whether cryoablation holds an advantage over RFA and MWA (23).

#### 2.1.1 Cryoablation and immuno-oncology in preclinical settings

In a murine model of prostate cancer, cryoablation resulted in an abscopal effect and reduced lung metastases, possibly by increasing the number of CD4^+^ and CD8^+^ T cells and natural killer (NK) cells (24). In another study involving a murine model of renal cell carcinoma (RCC) by Zhu et al., cryoablation combined with anti-PD-1 increased the percentage of CD8^+^ tumor-infiltrating T lymphocytes (TILs) and mRNA levels of interferon (IFN)-γ and granzyme B (GZMB) and inhibited distant tumor growth compared to cryoablation or anti-PD-1 alone (25). Similarly, the combination therapy also induced an effective abscopal effect in murine model of cervical cancer (26).

During this process, ICD releases DAMPs and tumor antigens, which activate immune cells mentioned above. The increased CD4^+^ T cells provide helper functions, facilitating the recognition and killing of tumor cells by CD8^+^ T cells and NK cells. Specifically, CD8^+^ T cells directly induce tumor cell lysis, while NK cells contribute to tumor clearance through mechanisms such as antibody-dependent cellular cytotoxicity (ADCC) (27). This immune response amplifies systemic immunity, potentially inducing an abscopal effect.

However, the immune response may vary in different tumor models. Yakkala et al. reported that cryoablation did not significantly contribute to systemic, antitumor, effector immune responses in a B16F10 melanoma model, and the function of immunotherapy is independent of cryoablation causing antitumor immune responses (28).

The application and long-term effects of cryoablation combined with ICI therapy must be further explored and verified in future preclinical studies.

#### 2.1.2 Cryoablation and immuno-oncology in clinical settings

##### 2.1.2.1 Hepatocellular carcinoma

Cryoablation is a safe and effective method that significantly improves the local control of primary HCC (>2 cm) compared to RFA or MWA (29).

Niu et al. reported that the combination of cryoablation with dendritic cell (DC)-cytokine-induced killer (CIK) cells (administered 3-5 days post-cryoablation, performed four times) extends the overall survival (OS) in patients with metastatic HCC compared to cryoablation alone (median OS, 32 vs. 17.5 months; *P* < 0.05) (30).

Lin et al. evaluated the clinical efficacy of cryosurgery combined with allogenic NK cell immunotherapy for advanced HCC and reported that the combined group exhibited better efficacy than the cryoablation group (median progression-free survival (PFS), 9.1 vs. 7.6 months; *P* = 0.0107) (31).

##### 2.1.2.2 Lung cancer

In addition to being a potentially curative and feasible treatment option for medically inoperable stage IA non-small-cell lung cancer (NSCLC) patients (32), cryoablation is safe and effective in patients with metastatic lung tumors (33, 34).

Feng et al. retrospectively analyzed cryoablation combined with anti-PD-1 antibody (nivolumab) treatment for advanced NSCLC and reported that patients in the cryo-nivolumab group had a significant improvement in immune function, and the total disease control rate (DCR) was 87.5% in the combined group versus 62.5% in the cryoablation group (*P* = 0.021) after 3 months of treatment (35); additionally, the levels of circulating tumor cells and tumor markers, such as cytokeratin 21-1 (CYFRA21-1) and neuron-specific enolase, were significantly reduced.

In the study by Nian et al., treatment with transbronchial cryoablation and anti-PD-1 antibody (camrelizumab) markedly reduced the tumor, and also improved obstructive pneumonia and atelectasis, demonstrating significant clinical efficacy in pulmonary sarcomatoid carcinoma (36).

##### 2.1.2.3 RCC

In RCC, cryoablation is most frequently used to treat clinical T1 cancer in patients who are not suitable for surgical resection (37).

Kato et al. reported increased frequencies of enriched T cell clones in the peripheral blood after cryoablation in RCC patients (38). In a pilot study on metastatic RCC, the immune cell infiltration of CD3^+^ and CD8^+^ T cells, CD20^+^ B cells, and PD-1^+^ immune cells were significantly increased in clear cell carcinoma patients treated with cryoablation combined with anti-CTLA-4 (tremelimumab) therapy compared to those treated with tremelimumab monotherapy (39).

Elevated levels of CD20^+^ B cells facilitate antibody production that mediates ADCC through NK cells, and present antigens to CD4^+^ T cells, thereby contributing to tumor cell clearance. The upregulation of PD-1^+^ immune cells augments T cell activation and promotes ICD. The synergistic activation of these immune populations not only enhances local tumor eradication but also holds significant potential for inducing systemic abscopal effects.

The study by Lin et al. showed that the combination of allogeneic NK cells and cryoablation had a synergistic effect in patients with advanced RCC by improving immune function and the objective response rate (ORR) was 80% in the cryo-NK group compared to 53.33% in the cryoablation monotherapy group after 3 months of treatment (40).

##### 2.1.2.4 Breast cancer

In breast cancer management, cryoablation has demonstrated remarkable efficacy across different disease stages. A phase II trial showed that cryoablation was effective in 92% of targeted lesions with 100% ablation in all breast tumors (size, <1.0 cm) (41), indicating its significant potential in early-stage disease. Furthermore, cryoablation of the primary tumor in patients with stage IV breast cancer is also reported to be safe and effective (42), suggesting the broad applicability of cryoablation as a treatment option across a spectrum of breast cancer stages.

In early-stage breast cancer, Comen et al. found that preoperative cryoablation combined with anti-CTLA-4 (ipilimumab) and nivolumab antibodies induced higher expression of T cell activation markers and serum Th1 cytokines; additionally, they reduced the number of immunosuppressive serum CD4^+^ PD-1^hi^ T cells and improved the effector-to-suppressor T cell ratios (43).

In metastatic breast cancer patients, Niu et al. retrospectively compared the effect of cryoablation plus immunotherapy of DC-CIK cells versus chemotherapy or cryoablation alone (44). Patients who received timely cryoablation (immediately after metastasis detection) combined with multiple cryoablations (administration of cryoablation therapy more than once) and concurrent immunotherapy demonstrated significantly prolonged median OS than the other groups.

##### 2.1.2.5 Melanoma

Cryoablation is generally not recommended for malignant melanoma because it may cause depigmentation and scarring of the treatment site, confounding future clinical and histological examinations (45). However, it is commonly used to treat melanoma metastases to slow the rate of tumor spread.

A pilot study demonstrated that the sequential therapeutic strategy combining cryoablation followed by intralesional administration of granulocyte-macrophage colony-stimulating factor (GM-CSF) at a standardized dose of 500 mcg exhibited well tolerability and efficacy in patients with unresectable metastatic melanoma (46). Hong et al. reported that cryoablation combined with anti-PD-1 (toripalimab) yielded a partial response in a patient with liver metastasis and advanced malignant melanoma (47).

Selecting the size of the intrahepatic lesion for cryoablation is important for the long-term survival of melanoma patients with liver metastases receiving anti-PD-1 therapy. A retrospective study by Shen et al. showed that patients with an intrahepatic tumor size of 15–45 mm and an ablated lesion size of ≤30 mm had significantly higher 3-month response rates (42.9% vs. 12.5%; *P* = 0.022) and survival times (30.5 vs. 14.2 months; *P* = 0.045) than their counterparts (48).

### 2.2 RFA

The first application of RFA was reported in the early 1990s for liver tumor (49). This method uses radiowaves of low frequency (460–480 kHz) and long wavelength to generate heat within a tumor mass. It induces cell death by direct thermal damage and coagulative necrosis, and the extent of tissue damage depends on the conductance of the tissue (50).

The focal zone temperature of RFA can typically reach between 60°C to 100°C. When the temperature reaches above 60°C, tumor cells begin to experience irreversible thermal damage. Temperatures between 70°C to 100°C can more effectively destroy tumor cells, as such high temperatures can lead to denaturation of intracellular proteins and disruption of the cell membrane. At the same time, heat diffusion creates a transitional zone (42°C–60°C) near the focal zone, where sublethal temperatures induce apoptotic cell death. This process, if accompanied with disruption of cellular membrane integrity, may transition into ICD. The temperature range of 42°C to 45°C is generally considered the starting point for cell damage, which may lead to impaired cell function but not necessarily immediate lethality. In the temperature range of 45°C to 60°C, tumor cells begin to experience thermal damage, and as the temperature approaches 60°C, the cumulative effect of cell damage becomes more pronounced, with the required temperature and time for therapeutic effect also decreasing accordingly (51, 52).

However, it is difficult to maintain the cytotoxic temperature if the ablated tumor is close to the great vessels. This heat-sink effect is a commonly described limitation of RFA and occurs when heat absorbed by flowing blood or air carried away from the ablation zone, thereby dissipating heat and reducing RFA effectiveness (53). Prolonged heating can lead to tissue charring, which can limit the further penetration of the ablative energy, and it may not penetrate as deeply into tissue as other ablation therapies, potentially leaving behind viable tumor cells.

Inflammatory infiltrates, including neutrophils, macrophages, DCs, NK cells, and ablated tissue-specific B and T cells, not only increased in the transitional zone, but also have been observed in distant untreated tumors and the peripheral blood in animals and patients (52). The immune changes of RFA are generally less than that of cryoablation, for example, in colon cancer murine model, cryoablation augmented secretion of a wider array of cytokines including interleukin (IL)-1β, IL-5, IL-6, and IL-10 than RFA, with both anti-tumor and anti-inflammatory profiles (54). The changes in peripheral T cell subsets following RFA appear to be independent of the tumor type or hepatitis status (55).

#### 2.2.1 RFA and immuno-oncology in preclinical settings

In a Lewis lung cancer (LLC) murine model, RFA removed the treated tumors and stimulated antitumor immunity, which could inhibit tumor growth in the non-ablated areas, melatonin is an important immunoregulatory molecule that can positively affect NK cells viability and further strengthen the killing effect of NK cells on target cells in a dose-dependent manner (56), the combined treatment of RFA and melatonin enhanced the RFA-stimulated NK activity and exerted synergistic antitumor effects with RFA (57).

In a study by Shi et al., RFA initially induced strong T cell-mediated immune responses in a murine model of colon cancer (CT26) and melanoma (B16), followed by the inhibition of the function of CD4^+^ and CD8^+^ T cells, driving a shift toward a higher regulatory T (Treg) cell to CD8^+^ effector T (Teff) cell ratio and up-regulating the PD-L1/PD-1 expression when combined with anti-PD-1 antibody (58). Liang et al. reported that the combination of RFA and anti-PD-L1 therapy increased CD8^+^T cell efficacy and decreased Treg infiltration in the murine model of HCC (59). Treg cells are capable of suppressing the activation of T effector cells and NK cells, inhibiting the maturation of DCs, and restraining the proliferation and differentiation of B cells. The reduction of Treg cells indicates that the combination is able to enhance the immune cycle, promote ICD, and elicits an abscopal effect.

#### 2.2.2 RFA and immuno-oncology in clinical settings

##### 2.2.2.1 HCC and metastatic liver tumors

RFA is commonly used to treat focal primary HCC and metastatic liver tumors, including those from primary colorectal and breast cancers (60).

A retrospective study involving recurrent HCC patients indicated that anti-PD-1 plus RFA had a better effect than RFA alone in improving the outcomes (median OS: 51 vs. 47.6 weeks, *P* = 0.008 and median recurrence-free survival (RFS): 39.1 vs. 19.3 weeks, *P* = 0.002) (61).

Ma et al. reported no severe adverse events, recurrences, or deaths during a seven-month follow-up in HCC patients with a tumor size of <4 cm after treatment with a combination of RFA and autologous RetroNectin activated killer cells (62).

##### 2.2.2.2 Lung cancer

RFA has demonstrated favorable safety and efficacy in patients with different stages of NSCLC (63). The combination of RFA followed by radical tumor resection (performed 8 days post-RFA) in early-stage NSCLC patients may induce an activated T cell-stimulatory phenotype in DCs, potentially enhancing long-term anti-tumor immunity against NSCLC (64). RFA combined with melatonin improves the clinical outcomes of early lung cancer patients with multiple pulmonary nodules; the combined treatment is associated with the suppression of non-ablated nodules and reduction of complications compared to surgery alone (57).

Yin et al. reported that RFA and a subsequent anti-PD-L1 antibody (atezolizumab) treatment exhibited a durable clinical benefit in a case of stage IV NSCLC (65). The patient initially underwent left upper lobectomy and then received 4 cycles of postoperative adjuvant chemotherapy. Six months following the completion of chemotherapy, a new lesion emerged in the left lower lobe, and RFA was performed. However, seven months after the ablation, the lesion in the left lower lobe enlargement, and new lesions emerged in both lungs, then the patient received atezolizumab. They found that the newly occurred left lung lesion which previously treated with RFA was responded to atezolizumab (administrate approximately 19 months after RFA), whereas the right lung lesion remained stable, indicating a significantly synergistic effect of RFA and subsequent immunotherapy.

### 2.3 MWA

Tabuse et al. first explored the application of MWA in treating liver cancer in 1986 (66). MWA uses heat generated by electromagnetic waves (frequency, 915 or 2450 MHz) to produce high temperatures (60–150°C), leading to tumor coagulation necrosis (67). It is associated with significantly reduced renal damage and is well tolerated in the case of large, inoperable lesions (68). MWA is less dependent on tissue characteristics, it can produce more accurate and larger ablation zones than RFA (69). The advancements in thermoacoustic imaging technology (70) will improve the effectiveness and application range of MWA. However, the ablation zones created by MWA can be less predictable and more spherical (71), which may not conform to the shape of irregularly shaped tumors. Although MWA is less affected by the heat sink effect than RFA, it still remains subject to this influence, especially around large blood vessels.

Compared to cryoablation and RFA, MWA induced relatively weak immune responses, with a significantly lower proinflammatory cytokine IL-1β and IL-6 production after MWA (72). Despite this, MWA combined with immunotherapy has emerged as a promising new direction in cancer therapy due to its technical feasibility, fewer side effects, and potential for synergistic effects in combination with immunotherapy, which can enhance the overall anti-tumor efficacy (73).

#### 2.3.1 MWA and immuno-oncology in preclinical settings

Sang et al. investigated the dynamic changes in the immune microenvironment of tumor-draining lymph nodes (TdLN) after MWA in an LLC murine model and reported a significant increase in the frequency of CD4^+^ T cells from day 1 to day 8 and a significant decrease in CD8^+^ T cell frequency on days 2 and 4 after MWA; however, no significant changes were observed on days 1 and 8 (74). In addition, a significant increase in IFN-γ secretion by NK cells was observed on day 4, whereas CD8 ^+^ T cells secreted IFN-γ and tumor necrosis factor (TNF) -α on day 8. This study implicated that the immune response elicited by MWA is limited.

Metal ions can continuously re-orient under the oscillating electromagnetic radiation and achieve efficient heating. Zhu et al. found that excess Ca^2+^ fixed with sodium alginate as Ca^2+^-surplus hydrogel could act as an efficient microwave-susceptible agent in a CT26 murine model, enabling enhanced microwave heating and inducing increased tumor ICD (15), thus indirectly promote immune effects. The study further demonstrated that the intratumoral fixation of Ca^2+^-surplus alginate hydrogel with Mn^2+^ ions could significantly improve the immune stimulatory effect of MWA treatment by activating the STING pathway.

In a murine colon cancer (MC38) model, Shao et al. reported that the combination of lymphocyte−activation gene 3 (LAG3) blockade and MWA promotes the proliferation and function of CD8^+^ TILs and transforms the tumor microenvironment (TME) into an antitumor state (75). In an MC38 and CT26 murine models, MWA combined with anti-PD-L1 treatment decreased tumor growth and prolonged OS, significantly suppressing CD8^+^ T cell depletion and enhancing their effector function; additionally, the levels of IFN-γ-stimulated transcription factors, particularly interferon regulatory factor 8 (IRF8), were also significantly increased (76).

Pan et al. reported that MWA combined with adoptive T-helper type 9 (Th9) cell transfer therapy significantly inhibited tumor growth, recurrence, and lung metastasis and ultimately prolonged the survival of NSCLC grafted mice (13); these effects were superior to those observed after MWA and Th9 cell transfer monotherapy. TME analysis showed that combined treatment significantly increased the number of tumor-infiltrating Th9 cells, enhanced the antitumor effect of CD8^+^ T cells, and remodeled the tumor immunosuppressive microenvironment and the tumor elimination efficacy in the mice models. Additionally, the superior effect of this combination therapy was demonstrated in humanized NSCLC patient-derived xenograft models in the same study (13).

The growth factor Flt3 ligand (Flt3L) plays a crucial role in the homeostasis and development of DCs by controlling their survival and expansion through binding to the Flt3 receptor tyrosine kinase on their surface. A recent study by Wang et al. showed that combining MWA with Flt3L significantly suppressed tumor recurrence by activating CD8^+^ T cells within the TdLN. When administered anti-PD-1 antibody with the MWA and Flt3L, the triple therapy significantly enhanced the tumor suppression. This offers a novel strategic approach for preventing tumor recurrence after MWA treatment (77).

#### 2.3.2 MWA and immuno-oncology in clinical settings

##### 2.3.2.1 HCC

MWA is a suitable option for early-stage HCC, with better prospects than RFA due to its higher thermal efficiency: enhanced tumor inactivation ability, reduced number of ablation sessions and fewer applications of puncture, a 60% decrease in the time required for ablation (78). MWA with parallel acupuncture guided by ultrasound is safe and effective for single HCC in high−risk areas (79). It is also increasingly becoming an option for the palliative treatment of advanced HCC (80).

Dong et al. analyzed inoperable HCC patients treated with MWA and found that the infiltration of T cells, NK cells, and macrophages increased significantly in both treated and untreated tumor tissues, improving patient survival (81). Zhou et al. reported that MWA may evoke a transitional immune response by increasing the frequency of Th17 cells in patients with hepatitis B virus−related HCC, while other circulating T−cell subsets may remain relatively stable after MWA (82).

Wang et al. investigated the synergetic antitumor impact of transfusion DC-CIK combined with MWA in Stage I/II HCC patients and reported a significantly longer disease-free survival with increased serum CD3^+^ (*P =* 0.049) and CD8/CD28^+^ (*P* = 0.045) levels compared to those seen after MWA alone (83). Li et al. used MWA in combination with apatinib and camrelizumab to treat patients with advanced HCC, which resulted in durable antitumor responses and significant improvements in PFS and OS compared to therapies involving a single drug or a two-drug combination in prior studies (84).

##### 2.3.2.2 Lung cancer

Compared to RFA, MWA typically produces a larger and spherical ablation zone, particularly in lung tumors, where the microwave field uniformly penetrates the lung tissue and is less dependent on its properties; alternatively, energy deposition and the effectiveness of RFA are often hampered and limited by the high electrical resistivity of the lung tissue (85). Li et al. reported that MWA spares the lung parenchyma, causing only a temporary decrease in pulmonary function; no difference in lung function was observed before and 1 month after MWA (86).

MWA is a safe, effective, and potentially curative therapy for patients with stage I NSCLC who are unsuitable for surgical resection (87). In patients with advanced NSCLC, combined MWA and chemotherapy resulted in longer PFS and OS than chemotherapy alone (88). Furthermore, MWA, as local consolidative therapy (performed for primary tumors and oligometastatic lesions before disease progression) after first-line epidermal growth factor receptor (EGFR)-tyrosine kinase inhibitors (TKIs) treatment, led to better disease control and survival than TKIs monotherapy in EGFR-mutant advanced NSCLC patients with extracranial oligometastases (89). Zhang et al. reported a reduction in Treg cells after MWA in patients with lung malignancies, which was independently associated with the PFS; however, an increase in the proportion of CD8^+^ T cells was noted (90). Xu et al. reported that IL-2 and IFN-γ levels in NSCLC patients treated with MWA decreased at 48 h and increased at 1 month post-ablation (91). The initial decrease in IL-2 and IFN-γ levels at 48h post-ablation may be a result of the body’s response to the injury caused by the ablation procedure, which temporarily suppress the immune system as the body focuses on healing the damaged tissue. The increased in these cytokine levels at 1 month suggests that the immune system is being activated. This could be a sign of the immune system recognizing the destroyed tumor cells as antigens and mounting an immune response against them, it implies that MWA could have immunomodulatory effects that enhance the body’s own ability to fight cancer (92).

Few studies have explored MWA in combination with ICI for advanced NSCLC. Wei et al. reported that combining MWA and camrelizumab improved the ORR to 33.3% in advanced NSCLC patients (93). In a retrospective study by Huang et al. involving stage III/IV NSCLC patients with EGFR/anaplastic lymphoma kinase (ALK)-wild-type, the combination of MWA and camrelizumab was reported to be safe and effective (94).

### 2.4 Irreversible electroporation (IRE)

Edd et al. first reported the results of *in vivo* experiments that confirmed the feasibility of IRE in 2006. IRE is an emerging tumor ablation technique that induces tumor cell apoptosis via high-voltage direct current to produce multiple nanoscale micropores on cell membranes without damaging peripheral structures and tissues, such as the blood vessels and nerves (95). However, the efficacy of the treatment can be limited by the depth of the electric field penetration, potentially in sufficient for larger or deeply seated tumors. Additionally, in complex anatomical areas, the precision of the electric field delivery may be challenging to achieve.

IRE leads to the exposure of many autologous tumor antigens *in situ* (96). It may elicit stronger immune responses than RFA as well as MWA, because the advantage of preserving blood vessels contributes to the transmission of immune molecules or cells.

#### 2.4.1 IRE and immuno-oncology in preclinical settings

Dai et al. showed that IRE significantly inhibited tumor growth in a murine model of HCC compared to untreated controls. Furthermore, the treated mice remained tumor-free after injecting secondary tumors (97). After IRE, an increase in CD8^+^ T cells was observed in the spleen and peri-ablation zones, and depletion of these cells led to local tumor regrowth and distant metastasis. Thus, the study indicated that CD8^+^ T cells were essential for IRE-mediated antitumor immunity.

Yang et al. demonstrated that IRE could overcome tumor-associated immunosuppression in a pancreatic cancer murine model (98). Furthermore, it could induce ICD, reduce the immunosuppressive components in the TME, and increase the infiltration of CD8^+^ T cells and GZMB^+^ cells when combined with DC vaccination (*P* = 0.001 and 0.007, respectively).

#### 2.4.2 IRE and immuno-oncology in clinical settings

The IRE ablation technique has been used to treat liver metastasis (in the proximity of the porta hepatis), RCC, localized prostate cancer, and lymph node metastases in locations where temperature-dependent ablation may be contraindicated (99–102). Its efficacy in pancreatic cancer is noteworthy. A median OS of 27 months could be achieved in patients with locally advanced pancreatic cancer (LAPC) (103), which is 4.5 times that seen in LAPC patients treated with general surgery.

Pan et al. reported that the serum levels of IL-2, TNF-β, and IFN-γ were significantly higher in LAPC patients after IRE ablation combined with NK cell therapy than in patients treated with IRE alone (*P* < 0.05) (104). Additionally, the combined treatment group exhibited significantly reduced levels of carbohydrate antigen 19-9 (CA19-9) at both7 days (*P* = 0.001) and 30 days (*P* = 0.019) after surgery, indicating a potentially greater efficacy in reducing tumor burden compared to IRE alone.

A retrospective study of LAPC patients treated with IRE plus toripalimab showed increased numbers of CD4^+^ and CD8^+^ T cells and higher levels of IL-4, IL-6, TNF, and IFN-γ, with prolonged OS (44.33 vs. 23.37 months, *P* = 0.010) and PFS (27.5 vs. 10.6 months, *P* = 0.036) compared with the IRE alone group (105). These findings indicated the potential survival and molecular benefits of IRE treatment in combination with anti-PD-1 antibody therapy.

In a multicenter study involving 200 patients with LAPC, adding IRE to conventional chemotherapy and radiotherapy resulted in substantial prolonged survival compared to that in the historical controls, with a median OS of 24.9 months (range: 12.4–85 months) in all patients (106).

### 2.5 High-intensity focal ultrasound (HIFU)

The effectiveness of HIFU on human localized prostate cancer *in vivo* was first reported in 1995 (107). It is a non-invasive, non-ionizing technique that uses focused ultrasound to produce areas of intense heat and cavitation, causing coagulative necrosis and mechanical cell damage. HIFU therapy for localized prostate cancer can achieve an oncologic control comparable to the conventional standard of care of, which includes radical prostatectomy and external beam radiation therapy, while reducing associated side effects caused by standard of care, such as incontinence and impotence (108). However, the penetration depth of HIFU is limited, which can make tumors located deep in the body less effective. Bone or air can scatter or absorb ultrasound energy, limiting the effectiveness of HIFU.

The immune response to HIFU is generally similar to RFA, but it can be influenced by the precise focusing and control of the ultrasound energy, potentially leading to a more localized immune reaction.

#### 2.5.1 HIFU and immuno-oncology in preclinical settings

In B16 melanoma and 4T1 breast cancer murine models, Cohen et al. demonstrated reduced infiltration and immunosuppressive activity of intratumoral Treg cells and myeloid-derived suppressor cells (MDSCs) following HIFU (109).

Mouratidis et al. reported prolonged survival, increased levels of CD8^+^ IFN-γ^+^ T cells, higher ratios of CD8^+^IFNγ^+^ T cells/Treg cells and CD8^+^IFNγ^+^/MDSCs, and activation of DCs after treatment with HIFU plus ICI in murine pancreatic tumors compared to the control untreated tumors and those treated with either HIFU or ICI alone (110).

MDSCs secrete immunosuppressive molecules, such as arginase-1 and inducible nitric oxide synthase, and promote the expansion of Treg cells. The reduction of MDSCs alleviates immunosuppression, allowing immune cells to attack tumors more effectively. Activated DCs enhance antigen presentation, promoting T cell activation and proliferation, and aid in the induction of ICD. Together, these factors facilitate an effective antitumor immune response and contribute to the generation of an abscopal effect.

#### 2.5.2 Immunomodulatory effect of HIFU in clinical settings

HIFU can achieve effective local tumor control with minimal treatment risks and side effects and has been used for various solid tumors, such as prostate cancer, breast cancer, and HCC (111–113).

Only relevant studies on HIFU eliciting immune responses in cancer patients are available; there are no clinical studies on HIFU plus ICI regimens in solid tumors to date. Wu et al. reported a significant increase in circulating CD4^+^ lymphocytes (*P* < 0.01) and the CD4^+^/CD8^+^ ratio (*P* < 0.05) in osteosarcoma, HCC, and RCC patients following HIFU treatment (114). In another study comprising 23 breast cancer patients, some tumor antigens remained in the tumor debris after HIFU ablation (115), which could act as a potential antigen source to stimulate the antitumor immune response.

### 2.6 Laser-induced thermotherapy (LITT)

In 1990, Sugiyama et al. first reported that LITT was safe and effective in treating deep brain tumors (116). The development of MRI thermometry introduced a breakthrough in the widespread use of LITT in neurosurgery. LITT refers to the selective ablation of lesions or structures using heat released by a laser with superior precision and predictable tissue ablation volumes, thus avoiding collateral damage (117).

LITT is particularly useful in cases where the tumor is located in a difficult-to-access location or in patients considered risky for surgery. Nevertheless, the limited penetration depth of the laser beam restricts its suitability for treating larger or deeply located tumors. Similar to RFA, extended exposure to the laser’s energy can result in tissue charring, thereby limiting the effectiveness of the treatment.

The immune response is comparable to RFA, it can cause blood-brain barrier (BBB) disruption, enhance antigen presentation, improve the function of cytotoxic CD8^+^ T cells, and exert an immunostimulatory effect on the TME (118).

#### 2.6.1 LITT and immuno-oncology in preclinical settings

LITT produced a significant and more prolonged heat shock proteins (HSP) 70 response in a murine model of colorectal liver metastases compared to the control untreated liver tissue, HSP70 can serve as a DAMP and enhance the activation of T cells; additionally, LITT induced a persistent increase in Kupffer cell activity in the liver and tumor tissues, which present tumor-associated antigens to T cells and secrete a variety of cytokines such as TNF-α, IL-1, and IL-6, which enhance the systemic immune response (119).

In another study, the combination of gold nanocar and LITT with anti-PD-L1 antibody reduced tumor growth, improved survival, and maintained a durable immune memory (rejection tumor rechallenge) in the CT-2A glioma murine model compared to those in the control group and monotherapy groups (120).

#### 2.6.2 LITT and Immuno-Oncology in Clinical Settings

LITT is mainly used to treat nervous system tumors, such as malignant glioma (121). It is also known to be effective in head and neck cancer, breast cancer, and lung cancer (122–124).

Paiva et al. reported prolonged survival in two patients with metastatic RCC in the head and neck who received a combination therapy of LITT plus IL-2 (125). Another case series by Hwang et al. reported partial responses and promising PFS and OS in two of three recurrent IDH-wild-type glioblastoma patients when treated with LITT plus immune anti-PD-1 antibody (pembrolizumab) (126). Nonetheless, well-designed trials with larger cohorts are needed to explore the potential of this combination.

## 3 Transarterial embolization (TAE) and immuno-oncology

Abnormal proliferation of tumor vessels is an important cause of tumor growth and spread. TAE can directly produce ischemic tumoricidal effects and induce tumor necrosis *in situ* by blocking the tumor blood supply, resulting in the production of tumor-associated antigens (127).

In 1983, Yamada et al. reported satisfactory results in 120 patients with unresectable liver cancer after TAE (128). With time, transarterial therapies have been more widely adopted to treat HCC (129). Embolization of metastases in patients, such as neuroendocrine tumors can provide adequate relief in those with carcinoid syndrome (130).

The main TAE methods include transcatheter arterial chemoembolization (TACE) and transarterial radioembolization (TARE). This section summarizes the effects of these approaches and their combined effects with immunotherapy. The ongoing trials of TAE combined with immunotherapy are shown in **Table 2**.

**Table 2:** Ongoing trials of transarterial embolization combined with immunotherapy.

NCT number	Enrollment	Phase/N	Design	Primary Measures	Study Completion
NCT04472767	HCC	Phase 2 N = 35	TACE + Cabozan + Ipi + Nivo	6-month PFS 1-year CR rate	September, 2027
NCT05776875	HCC (BCLC B)	Phase 2 N = 24	TACE + Atezo + Beva	2-year AEs (grade ≥ 3)	August, 2025
NCT03937830	HCC or BTC	Phase 2 N = 72	TACE + Durva + Beva + Treme Durva + Beva + Treme	6-month PFS	December, 2025
NCT05751343	Unresectable HCC	Phase 2 N = 55	TACE-HAIC + Atezo + Beva	1-year ORR	February, 2025
NCT04268888	Intermediate-stage HCC	Randomized Phase 2/3 N = 522	TACE/TAE + Nivo TACE/TAE	2-year OS 2-year TTTP	June, 2026
NCT05185505	HCC beyond MC to transplantation	Phase 4 N = 24	TACE + Atezo + Beva	1-year acute rejection proportion	October, 2027
NCT06503250	Unresectable HCC	Observational N = 150	TACE + Atezo + Beva	1-year real-world PFS	September, 2025
NCT06323382	Advanced HCC	Observational N = 240	TACE/HAIC + PD-1/PD-L1 inhibitor + Beva	1-year PFS	December, 2024
NCT05717738 (CCGLC-008)	Advanced HCC	Observational N = 300	TACE + Len/Sor/Don/Reg + PD-1 inhibitor TACE + Atezo + Beva TACE + Beva + Sint TACE + Apa + Camre	3-year number of patients amendable to curative surgical interventions	December, 2024
NCT06607120	Advanced HCC	Observational N = 950	TACE + PD-1/PD-L1 inhibitor + VEGF-TKI/Beva PD-1/PD-L1 + VEGF-TKI/Beva	2-year OS	March, 2025
NCT06058663	Intrahepatic cholangiocarcinoma	Phase 1 N = 18	TARE + Treme (day 1) + Durva TARE + Treme (day 14) +Durva	Day 0–28 AEs Day 14–42 AEs	November, 2025

HCC, hepatocellular carcinoma; TACE, transarterial chemoembolization; Cabozan, cabozantinib; Ipi, ipilimumab; Nivo, nivolumab; PFS, progression-free survival; CR, complete rate; BCLC B, Barcelona Clinic Liver Cancer Stage B; Atezo, atezolizumab; Beva, bevacizumab; TACE, transarterial chemoembolization; AEs, adverse events; BTC, biliary tract carcinoma; Treme, tremelimumab; HAIC, hepatic artery infusion chemotherapy; ORR, objective response rate; TAE, transarterial embolization; OS, overall survival; TTTP, time to TACE progression; Durva, durvalumab; MC, milan criteria; PD-1, programmed death-1; PD-L1, programmed death-ligand 1; VEGF-TKI, vascular endothelial growth factor tyrosine kinase inhibitor; Len, Lenvatinib; or, sorafenib; Don, donafenib; Reg, regorafenib; Sint, sintilimab; Apa, apatinib; Camre, camrelizumab; TARE, transarterial radioembolization.

### 3.1 TACE

TACE is a minimally invasive interventional therapy in which a drug-loaded embolic agent is injected directly into the tumor-feeding artery to produce locoregional chemotherapy and embolization at the tumor site synergistically.

It can be performed with lipiodol (conventional, cTACE) or drug-eluting beads (DEB-TACE). DEB-TACE was reported to improve the drug loading capacity by absorbing high concentrations of electropositive chemotherapeutic agents; however, it did not appear to have an advantage over cTACE in terms of efficacy (131).

A systematic review of randomized trials involving unresectable HCC showed that TACE significantly improved the OS in comparison to the control group of conservative management (132). It is the first-line therapy for treating intermediate-stage B HCC, according to the Barcelona Clinic Liver Cancer Staging System (133, 134).

There are conflicting opinions on the effects of TACE on immune regulation. On the one hand, TACE promotes the release of tumor antigens and proinflammatory cytokines, promotes ICD, and converts non-immunogenic tumors into immunogenic tumors (135, 136). Ren et al. demonstrated that the Treg cell proportion after TACE was significantly lower in HCC patients, which indicated the positive regulatory effect on the anticancer immune function of HCC patients (137). On the other, hypoxia of residual tumor cells caused by incomplete embolization up-regulates vascular endothelial growth factor (VEGF) expression, which hinders DC maturation and function and increases the recruitment of Treg cells and MDSCs, resulting in immunosuppressive effects (138, 139). Hypoxia is an important cause of tumor recurrence and metastasis in HCC after TACE, by promoting tumor cell invasiveness, angiogenesis, drug resistance, metabolic changes, and the maintenance of tumor stem cell characteristics.

Therefore, the efficacy of TACE may be increased when combined with anti-VEGF antibody and ICI. A prospective, single-arm, phase 2 study of TACE combined with anti-PD-L1 antibody (envafolimab) and lenvatinib in unresectable HCC demonstrated promising survival outcomes and operable conversion rates with a tolerable safety profile (140). A global, open-label, phase 3 IMbrave150 trial established that treatment with atezolizumab plus anti-VEGF antibody (bevacizumab) resulted in better OS and PFS outcomes than sorafenib in patients with advanced HCC (141). Another phase 3 EMERALD-1 trial demonstrated that the combination of TACE with anti-PD-L1 antibody (durvalumab) and bevacizumab significantly improved the PFS (median, 15 vs. 8.2 months; HR (hazard ratio), 0.77; *P* = 0.032) in unresectable HCC patients compared with TACE alone; the PFS after treatment with TACE plus durvalumab was not significant (median: 10 vs. 8.2 months, HR 0.94, *P* = 0.638) (142).

A nationwide multicenter, retrospective cohort study comprising patients with advanced HCC, showed that first-line therapy with TACE plus ICI plus anti-VEGF antibody was associated with significantly longer OS (median, 22.6 vs. 15.9 months; HR, 0.63; *P* < 0.0001), PFS (median, 9.9 vs. 7.4 months; HR, 0.74; *P* < 0.0001), and ORR (41.2% vs. 22.9%; *P* < 0.0001) and acceptable safety profiles than treatment with ICI plus anti-VEGF antibody (143). This finding indicates that TACE plays an important role in the TACE-ICI-VEGF regime, which may be due to the effective reduction of the intrahepatic tumor burden by TACE, thereby improving the efficacy of ICI-VEGF.

Nanomaterials have emerged as a promising strategy in cancer immunotherapy with the advent of nanotechnology (144). Nanovaccines contain tumor antigens to which T cells specifically respond, immune adjuvants that enhance immune responses, and targeted delivery vaccine systems, such as ligand-directed targeting, molecular targeting, and pH-sensitive vesicles, which improve the sensitivity of immunotherapy (145). Shi et al. designed a nanovaccine that can synergize TACE, induce ICD of tumor cells under hypoxia, increase the infiltration of primary and distant tumor-toxic T lymphocytes, promote the maturation of DCs in lymph nodes, and activate potent antitumor immune responses (14). It improves hypoxia and immunosuppressive TME after TACE and prevents tumor recurrence and metastasis. In the long-term, sustained progress in biomimetic nanovaccines will help enhance existing technologies and potentially revolutionize the clinical landscape for cancer management.

The efficacy of TACE is severely limited by the inferior drug burst behavior, which is typically due to factors such as poor drug carrier design, material properties, and the insufficient biocompatibility, resulting in a lack of long-term drug release controllability, which affects its immune response (146). Ma et al. developed gelatin-based drug-eluting microembolics grafted with nanosized poly (acrylic acid), which exhibited vessel remodeling-induced permanent embolization with minimal inflammatory responses after complete degradation (147). This resulted in an effective and versatile strategy for enhancing the long-term therapeutic responses of various local chemotherapy treatments. Furthermore, the microembolics demonstrated optimal mechanical, pharmaceutical, and biological properties, with excellent micro-catheter deliverability in a healthy porcine liver model, and significantly augmented the TACE efficacy in a rabbit VX2 liver cancer model (147).

### 3.2 TARE

TARE uses yttrium-90 (Y90), administered via resin or glass microspheres. Local radiation causes direct DNA damage and tumor cell death by injecting radioactive microspheres into the hepatic artery (148). However, TARE is associated with radiation-related toxicity, and compared to TACE, its therapeutic effects typically manifest more slowly, which may be disadvantageous for rapidly progressing diseases.

TARE can be used to treat HCC and other primary and secondary hepatic malignancies, such as intrahepatic cholangiocarcinoma and hepatic metastases from neuroendocrine cancer, ocular melanoma, and breast cancer (149). The irradiated tumor cells affect the tumor microenvironment and may act as an immunogenic hub, inducing an abscopal effect. Chew et al. compared peripheral blood mononuclear cells before and after TARE and observed an infiltration of GZMB ^+^ cells, CD8^+^ T cells, CD56^+^ NK cells, and CD8^+^ CD56^+^ natural killer T (NKT) cells, with increased TNF-α levels and antigen-presenting cells after TARE (150). GZMB ^+^ cells can release GZMB, which primarily participate in cytotoxic immune responses and can induce apoptosis of tumor cells (151). Among NK cells, CD56^+^ NK cells typically possess a more effective ability to kill tumor cells and are also more prone to secreting cytokines such as IFN-γ and TNF-α (152). CD8^+^ CD56^+^ NKT cells possess both the cytotoxicity of T cells and the regulatory functions of NKT cells, they can recognize and eliminate tumor cells, while also regulating the immune response through the release of cytokines (153), thereby enhancing the tumor immune cycle, inducing ICD, and further eliciting an abscopal effect.

Next-generation sequencing confirmed the upregulation of genes involved in innate and adaptive immune activation in TARE-treated tumors. Young et al. evaluated 102 TARE treatments in 93 patients with HCC. Their findings revealed that patients with pretreatment absolute lymphocyte counts exceeding 1 × 10^3^/μL pretreatment demonstrated significantly longer OS at 1, 3, and 6 months post-treatment compared to those with counts below this threshold (154).

Deipolyi et al. analyzed the immune effect of TARE on liver metastases from breast cancer to assess the clinical utility of PD-1 levels (155). The clinical response was associated with an increase in baseline PD-1 expression by CD4^+^ TILs in the TME, highlighting the potential synergistic effect of TARE and anti-PD-1 antibodies in treating liver tumors. A retrospective case series of 11 patients with uveal melanoma-derived unresectable hepatic metastases demonstrated that TARE combined with anti-CTLA-4/PD-1 antibody is safe and effective (156). Another single-center retrospective study comprising 26 patients with HCC who received TARE with nivolumab and/or ipilimumab revealed a median OS of 16.5 months and PFS of 5.7 months from the first TARE, thus indicating that the combined regimen was safe with limited treatment-related toxicity (157).

These results promote prospective studies evaluating the combined regimen of TARE plus ICI for HCC treatment. In a Phase 1 clinical trial involving HCC patients, the disease control rate was 82% (N = 9/11) in the TARE plus nivolumab group (158). A Phase 2 trial of 36 patients with advanced HCC who received TARE followed by nivolumab showed an ORR of 30.6% (159). Further exploration is needed to determine the optimal time for administering the combined regimen.

## 4 Future perspectives and challenges

IO emerges as a dynamic field at the forefront of medical innovation, reshaping the landscape of cancer care. The evolution of image-guidance technologies, particularly the emergence of real-time multimodal fusion imaging, has significantly advanced IO (7). For example, the recent innovations in thermoacoustic imaging will enhance the therapeutic precision and clinical applicability of MWA through improved tumor margin delineation and energy deposition monitoring (70). Nonetheless, the issue of recurrence persists, such as the recurrence of HCC at the margins of the ablation zone. To reduce the risk of recurrence due to microscopic invasion at the tumor periphery, it is recommended to create an adequate tumor-free margin. However, in HCC patients with cirrhosis or tumors in locations at risk, the extension of the ablation boundary is often limited to protect liver function and surrounding critical tissues. Faced with this challenge, three-dimensional imaging guidance and navigation techniques will help achieve adequate ablation margins. Moreover, for tumors at risk sites, the combination of temperature monitoring and low-dose ethanol injection can achieve relatively favorable local tumor control. Ensuring optimal needle positioning by trained teams on the first pass that minimizes the number of insertions is also a crucial factor in preventing recurrence (160).

Over the past 20 years, learning-based algorithms using artificial intelligence (AI) have emerged in medical imaging research and will be essential for IO clinical practice (161). The synergistic alliance between human proficiency and technological advancements propels IO into an era of unprecedented possibilities. AI-driven models based on thousands of previously treated patients predict the selection of an individualized treatment modality and may be associated with the best local tumor control rate and the lowest chances of complications. The mantra “cheaper, better, faster” has long been used to generalize IO, and providing effective, cost-efficient, and safe treatments with decreased side effects will remain the cornerstone of the specialty.

With the advent of ICI, the therapeutic efficacy of IO can be pushed to a new horizon. Subsequently, the innovative concept of interventional immuno-oncology emerged, synergistically integrating the principles of IR and immuno-oncology to revolutionize cancer treatment. This groundbreaking approach not only demonstrates enhanced anti-tumor efficacy but also represents a significant advancement in oncology, offering patients novel therapeutic options and comprehensive treatment strategies in the battle against cancer. However, the treatment effect varies among patients; hence, we need biomarkers to predict the immune status, tumor characteristics, and potential response to the therapy. Pre-treatment biomarkers play a crucial role in identifying patients most likely to benefit from ICI and predicting the immune response to the therapy. The high PD-1/PD-L1 expression, tumor mutational burden, and immune cell infiltration associated with a potential for stronger immune response may guide therapeutic decisions. Post-treatment biomarkers, such as circulating immune cells that secret cytokines, can evaluate the effect of the treatment. For example, the increased proportion of CD8^+^ T cells and high levels of IFN-γ after intervention may indicate that including ICI could enhance the positive immune response (162). Moreover, the time of ICI use may be guided by the immune change period. For example, MWA of lung cancer can modulate the immune microenvironment within the TdLN, initiating an immune response. Notably, the alterations are particularly evident and intricate during the first 4 days following MWA, ICI combined with MWA within a certain period of time may enhance anti-tumor immunity (74).

Cancer treatment will be more individualized and precise in the future. TME cells and their secreted molecules are considered to play critical roles in cancer pathogenesis. The TME is complex, with numerous interconnections and variations among different organs and patients (163). The diversity of the TME may be attributed to the different immune responses caused by the same treatment modality. On the one hand, IO can enhance the local and systemic immune responses; on the other, the same treatment can inhibit the immune response. For example, when cryoablation combined with ICI in the RCC and cervical cancer murine models, it contributes to effector immune responses, inhibited tumor growth and induced an effective abscopal effect (25, 26). But in a B16F10 melanoma model, the synergistic immune response was not found (28). TARE can activate the immune response (150) but may also result in lymphopenia and immune system suppression through chronic inflammation (164), adversely affecting the outcome. Thus, the key question now is how to combine IO and ICI in a rational and optimal manner. For preclinical studies, lack of orthotopic models is a concern for most deep-seated solid tumors. Qi et al. generated an experimental RFA platform with orthotopic murine model of HCC and a readily available human radiofrequency generator, it better simulate the actual conditions within the human body and aids the study of mechanisms of RFA in HCC (165). We hope that more of these orthotopic murine models will be built to better investigate the TME, all the complexities of TME will be analytically integrated in future studies and that the best treatment modality will be identified.

## 5 Conclusion

Interventional therapy can effectively promote local tumor control and release new antigens in the tumor site. The cancer intervention criteria may be redefined with advancements in AI, imaging modalities, and personalized medicine, leading to better patient outcomes. The combination of interventional therapy and immunotherapy demonstrates a synergistic effect through modulation of immune cell function, but may concurrently induce immunosuppressive effects. Much efforts are still required to elucidate the mechanisms by which combination regimens modulate immune system responses, which will provide critical insights for optimizing the integration of interventional and immunotherapeutic strategies.
